# Genetic Analysis of Walnut (*Juglans regia* L.) Pellicle Pigment Variation Through a Novel, High-Throughput Phenotyping Platform

**DOI:** 10.1534/g3.120.401580

**Published:** 2020-10-02

**Authors:** Gina M. Sideli, Peter McAtee, Annarita Marrano, Brian J. Allen, Patrick J. Brown, Timothy S. Butterfield, Abhaya M. Dandekar, Charles A. Leslie, David B. Neale

**Affiliations:** * Department of Plant Sciences, University of California, Davis, CA 95616; † Plant and Food Research, Auckland, 1142, New Zealand; ‡ U.S. Department of Agriculture, ARS Crops Pathology and Genetics Unit, Davis CA 95616

**Keywords:** computer vision system, walnut, seed coat, bi-parental population, genome-wide association

## Abstract

Walnut pellicle color is a key quality attribute that drives consumer preference and walnut sales. For the first time a high-throughput, computer vision-based phenotyping platform using a custom algorithm to quantitatively score each walnut pellicle in L* a* b* color space was deployed at large-scale. This was compared to traditional qualitative scoring by eye and was used to dissect the genetics of pellicle pigmentation. Progeny from both a bi-parental population of 168 trees (‘Chandler’ × ‘Idaho’) and a genome-wide association (GWAS) with 528 trees of the UC Davis Walnut Improvement Program were analyzed. Color phenotypes were found to have overlapping regions in the ‘Chandler’ genetic map on Chr01 suggesting complex genetic control. In the GWAS population, multiple, small effect QTL across Chr01, Chr07, Chr08, Chr09, Chr10, Chr12 and Chr13 were discovered. Marker trait associations were co-localized with QTL mapping on Chr01, Chr10, Chr14, and Chr16. Putative candidate genes controlling walnut pellicle pigmentation were postulated.

English walnut (*Juglans regia* L.) is an economically important edible seed that is diploid 2x = 32 with 16 chromosomes and a genome size of approximately 600 Mbp. In the U.S., the English walnut industry generates 1.5 billion dollars annually with over 99% of production occurring in California (CA Dept Food Ag 2018). Interestingly, consumer preference is for a lighter color seed coat, or pellicle, thereby commanding higher prices in the U.S market.

The seed coat, pellicle and/or testa covers the zygotic embryo (seed) and is formed from maternally derived tissue arising from the inner and outer ovular integuments (Mohamed-Yasseen *et al.* 1994). Each seed coat layer contributes to various metabolic functions which include: producing, transporting, and accumulating metabolites; photosynthetic assimilates for zygote development; defense; and physical structure (Smýkal *et al.* 2014).

Walnut (*Juglans regia* L.) pellicle pigmentation can vary significantly between cultivars and developmental stages, and is influenced by environmental stressors. Flavonoids or phenolic compounds synthesized through the phenylpropanoid pathway are localized in seed coats to defend the embryo against biotic stressors (Dixon *et al.* 2013), and generally contain yellow pigments.

Currently, pellicle color is scored qualitatively on an ordinal scale of 1 – 4 (extra light, light, light amber, amber), and subjectively by eye using a color chart published by the California Dried Fruit Association (DFA) (Figure 1A). Color perception by the human eye is complex and has been researched since at least 1878 when Hering, a physiologist proposed the opponent color theory as function of both psychological and neurological processes and Hunter in 1979 explained that receptors in human eye perceive color in pairs (Lee 2008). In particular, L* is the scale of light to dark, a* is the scale of green to red, and b* is the scale of blue to yellow (Hunter and Harold 1987) (Figure 1B). The Lab color scale is arguably one of the most perpetually uniform color spaces and best approximates color difference as perceived by the human eye.

**Figure 1:**
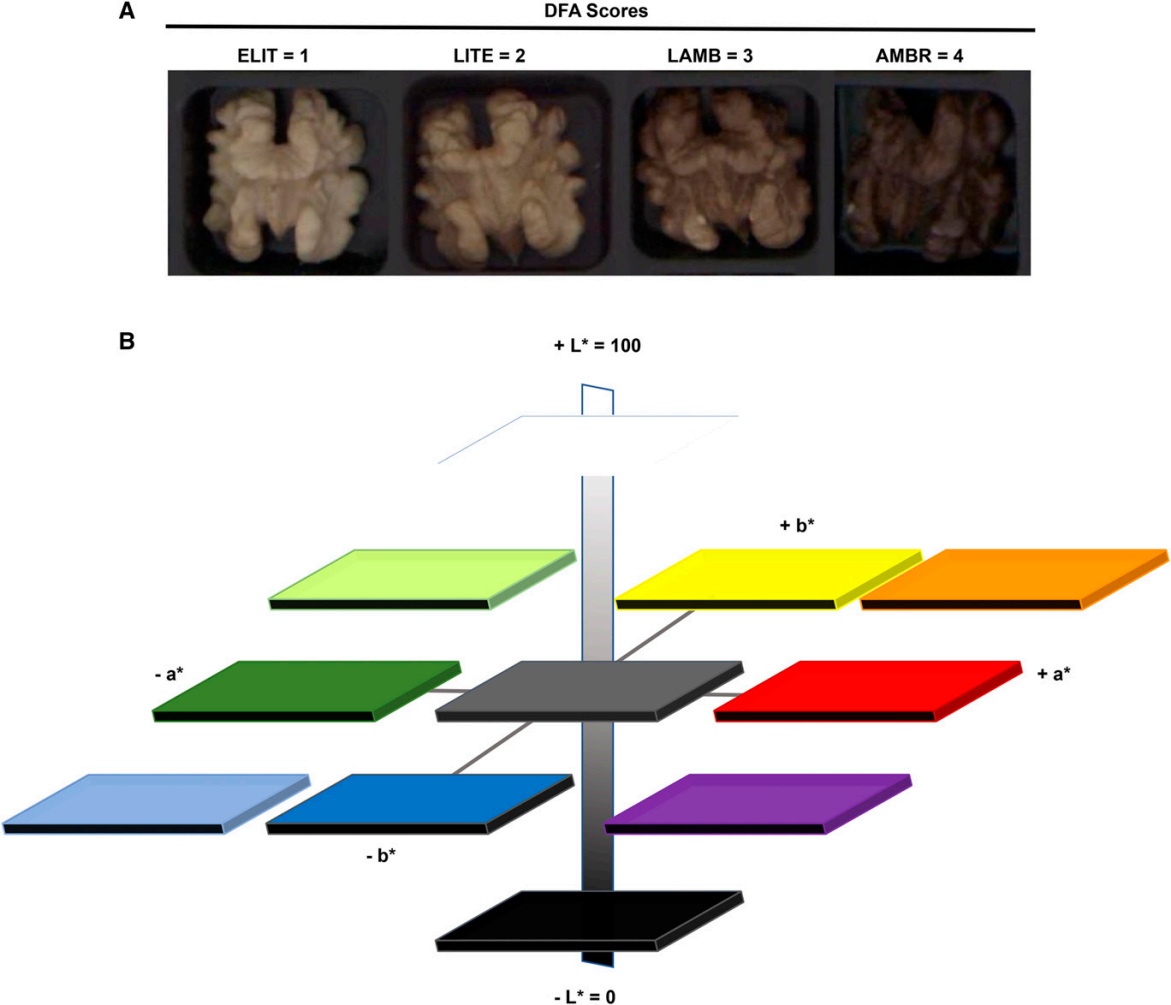
Qualitative *vs.* Quantitative scoring of walnut pellicle. A. Qualitative scoring according to the DFA method. ELIT= Extra light, LITE = light, LAMB = light amber, AMBR = amber. B. Hunter L* a* b* color scales in a three-dimension representation based on color opponent theory. L*measures the degree of black to white where a low number (0-50) indicates dark, and a high number (51-100) indicates a lighter color, a* measures the degree of red (positive number) to green (negative number), and b* measures the degree of yellow (positive number) to blue (negative number).

Human perception of color is largely influenced by the number and types of photoreceptors an individual possesses. Differences in the photoreceptors between individuals contributes to variability in the perception of a given color. This forms the basis of subjectivity of human quality assessments within a color perception dataset. As such, color(s) quantified by human perception can be an unreliable source for obtaining quality data (Brosnan and Sun 2004), however since the trait is influenced by consumer acceptance, human perception cannot be ignored completely. Computer vision systems (CVS) have been proposed as a large-scale replacement to quality assessments by human users. A CVS is any platform that acquires and processes images with computer software by using a algorithm for calibration and color extraction. The CVS interprets color in Hunter’s L* a* b* space (Figure 2) by using a method adopted by Leon 2006 where pixels in RGB from an image are fit in a linear model and transformed to Lab scores. This is vastly more high-throughput and consistent than a human user qualitative score. Computer vision systems have been used in a variety of crops for quality grading in carrot (Deng *et al.* 2017), detection of mechanical damage in blueberry (Wang *et al.* 2018), table grape rachis browning (Bahar *et al.* 2017), and sweet lemon mechanical damage destruction (Firouzjaei *et al.* 2018).

**Figure 2:**
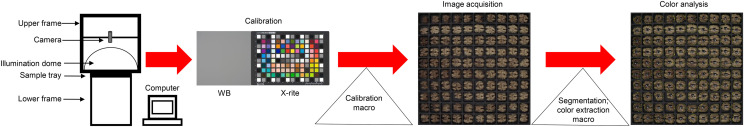
Computer Vision System processing schematic. Computer vision system consists of a box frame with an attached camera fixed over an illumination dome enclosed on all four sides. Performed steps are 1)image acquisition of WB-white balance and color checker 2)calibration macro 3)image acquisition of pellicles 4) processing macro which segments kernels from background and extracts quantitative color scores.

Using traditional scoring, walnut pellicle color is labor intensive to measure, strongly influenced by environmental variation, and is a polygenic trait (Marrano *et al.* 2019). Combining quantitative phenotypic measurements of a CVS with genotyping data will facilitate identification of genetic loci that explain trait variation. Causal single nucleotide polymorphisms (SNPs) can be validated with marker-assisted breeding or genomic prediction models. This makes it possible to identify desirable parents and select preferred progeny at the young seedling stage rather than at sexual maturity 4-5 years post-germination. Substantially reducing the seedling-selection time will hasten the development of new cultivars, decrease costs in allocation of resources, and accelerate the genetic gain.

The first objective of this study was to measure walnut pellicle color quantitatively using a CVS developed by (Donis-González *et al.* 2020) that discriminates color in the L* a* b* channels. The second objective was to use quantitative-trait loci (QTL) mapping and genome-wide association study (GWAS) to detect marker trait associations combined with SNP genotyping.

## Materials and Methods

### Walnut source and experimental design

The experiment was performed with *Juglans regi*a seedling trees ranging from age 4 – 12 which were derived from multiple crosses within the UC Davis Walnut Improvement Program.

The genome wide association design consisted of seedling trees from 31 full-sib families with an average of 16 per family. These trees ranged in age from 4 – 9 years, and included 20 founder trees, and one cultivar ‘Robert Livermore’ released by UC Davis for a total of 528 trees. A bi-parental F1 population ‘Chandler’ × ‘Idaho’ (n = 168) was used for QTL mapping with a range in tree age of 8 – 12. Trees in this study were planted across eight blocks; five of them were across the same field location and all trees were spaced six feet apart and irrigated with micro-sprinklers. Blocks consisted of trees planted in row plots with multiple family crosses made in one year. A full-sib family had between 10 and 50 individuals with 31 unique parents. Most male parents were only used once, with a few male parents used 2-4 times, one male was used for 13 different crosses, and two males were used reciprocally as females.

At least 30 nuts per tree were hand harvested as each tree reached fruit maturity (hull-split) during August and September of 2015, 2016, and 2017. These were hung in polypropylene mesh bags, air-dried for two weeks, and then stored at -4° until measurements were taken. Walnut kernels were placed on commercial scoring trays obtained from the Dried Fruit Association of California which contain 100 wells each. Each tray held ten kernels from each of 10 distinct trees, and 10 kernels were evaluated. When kernels were being evaluated for color, they were kept at 0° for short-term storage, and kernels were kept at - 4° for long term storage.

### Measuring pellicle color

Qualitative color scoring by eye was done by examining each kernel for its separation into color category 1 - 4, according to the Dried Fruit Association (DFA) scoring sheet (Figure 1A), (Safe Food Alliance, Sacramento, CA, (DFA of California 2017), in accordance with the USDA Standards for Grades of Shelled Walnuts as designated by the California Walnut Board under Title 7 of the United States Code (U.S.C.) (7 C.F.R. § 51.2277-2296).

### The DFA color scores are

ELIT = Extra light, 1^a^; LITE = light, 2^b^; LAMB = light amber, 3^c^; AMBR = Amber, 4^d^ (Figure 1A.)

^a^Extra light permits a 15% tolerance for kernels darker than “Extra Light”.
^b^Light permits a 15% tolerance for kernels darker than “Light”.
^c^Light Amber permits a 15% tolerance for kernels darker than “Light Amber”.
^d^Amber permits a 10% tolerance for kernels darker than “Amber”. Darker than “Amber” is black.

Pellicle color of each kernel was scored by visual inspection according to the DFA color chart for 10 kernels per tree and the mean DFA was calculated. This was repeated for each harvest year.

The computer vision system was developed with a Basler 5.5 megapixel camera (Ahrensburg, Germany) which mounts over an enclosed dome encircled with four (D65) light emitted diodes (LED) tubes of 18W (Phillips, Amsterdam, Netherlands) which provided consistent illumination. Custom algorithms were developed for calibration and image acquisition of the computer vision system (File S1, File S2). Briefly, The calibration macro written in FIJI 1.52 software (Schindelin *et al.* 2012) divided a white balance WB image into three color channels RGB, and subtracted the maximum value of each channel according to a color checker standard. The CIE color analysis macro written in FIJI 1.52 software (Schindelin *et al.* 2012), enabled acquired images of walnut pellicles in 100 welled trays, in a JPEG format, to perform batch runs with output measurements of color in L* (lightness), a* (red/green color), and b* (yellow/blue color). The data produced contains each color scores for every pellicle measured. Details on system and method was modified from (Donis-González *et al.* 2020) to enable segmentation of both light and dark kernels from tray background, run much faster at less than 30 sec per image, and run silently in computer background without a region of interest (ROI) manager pop-up (Figure 2, File S3).

### Analysis of phenotypic variation

Multi-year phenotypic data collected for DFA scores and the CVS scores were analyzed with ANOVA using R packages ‘car’, ‘agricolae’, ‘lme4’ with equation (1) for the model,

$${\text{Y}}_{\text{ijkl}}=\text{μ}+f_{\text{l}}+g_{\text{i}}+y_{\text{k}}+{\left(fy\right)}_{\text{ik}}+a_{\text{ijk}}+{\text{ε}}_{\text{ijkl}}$$ (1)

where Y is the color measurement taken on the i-th genotype, with j number of measurements in the k-th year with l number of families; μ is a constant value (overall mean), *f*_l_ is the effect on the color measurement of the l-th family, *g*_i_ is the effect on color measurement of the i-th genotype, *y*_k_ is the effect on color measurement of the k-th year, (*fy*)_lk_ is the interaction between family and year, *a*_ijk_ is the effect of age on color measurement for the i-th genotype, j number of measurements in the k-th year, and ε_ijkl_ is the random error term with mean 0 and variance σ^2^_ε_.

For the ‘Chandler’ × ‘Idaho’ population, the adjusted means were calculated in R package ‘lsmeans’ to account for the year differences and one value per individual tree was used in the QTL analysis with the equation (2) for linear model,

$${\text{Y}}_{\text{ijk}}=\text{μ}+{\text{α}}_{i}+{\text{β}}_{k}+{\text{(αβ)}}_{\text{ik}}+{\text{ε}}_{\text{ijk}}$$ (2)

where y is the color measurement taken on the i-th genotype, with j number of measurements in the k-th year, α_i_ is the effect on color measurement of the i-th genotype, β_k_ is the effect on color measurement of the k-th year, (αβ)_ik_ is the interaction between genotype and year, and ε_ijk_ is the random error term with mean 0 and variance σ^2^_ε_. For the larger dataset of the 31 full-sib families, breeding values (BV) per individuals were estimated using ASReml-R v4 (Butler *et al.* 2017) in order to remove environmental effects of years and blocks on the phenotypes. In particular, the mixed model for equation (3),

y=Xτ+Zu+e (3)

where y denotes the (n × 1) vector of observations, *τ* is a (p × 1) vector of fixed treatment effects (block and year), *X* is an (n × p) design matrix, u is the (q × 1) vector of random effects (pedigree), *Z* is an (n × q) design matrix, e is the (n × 1) vector of residual error. The pedigree utilized in the model was reconstructed to identify corrected kinship relationships from the original UC Davis Walnut Improvement Program pedigree using the Axiom *J. regia* 700K SNP array (Marrano *et al.* 2018). Narrow sense heritability per trait was estimated with equation (4),

$$h^{2}=\frac{\sigma_{A}^{2}}{\sigma_{P}^{2}}=\frac{\sigma_{A}^{2}}{\left(\sigma_{A}^{2}\right)+\left(\sigma_{R}^{2}\right)}$$ (4)

where the additive genetic variation $\sigma_{A}^{2}$ is divided by the total phenotypic variation $\sigma_{P}^{2}$ (additive + environmental variation or residual).

### QTL mapping

Quantitative-trait loci mapping was performed using the genetic maps (File S5) of the bi-parental population ‘Chandler’ × ‘Idaho’ described in (Sideli *et al.* 2020). In particular, the ‘Chandler’ genetic map spanned 998.31 cM with 1,165 markers in total, while the ‘Idaho’ map contained 1,753 markers for a total length of 1,693.88 cM. All measurements of pellicle color were evaluated in R/QTL software v1.44.9 (Broman *et al.* 2003). Phenotypic measurements from each year and the adjusted mean of the two years were first assessed using simple interval mapping in a single QTL model with *scanone* and ‘Haley Knott’ regression (Haley and Knott 1992). Significance thresholds were defined using 1,000 permutations for estimating critical values of alpha = 0.05 (Churchill and Doerge 1994). When multiple QTL were detected per trait, their precise location was adjusted and tested using ANOVA by considering both QTL locations. Briefly, utilizing the function *sim.geno*, multiple imputations were performed to fill in missing genotype data with a step of 1 (cM) and 100 imputations. A multiple QTL model was then fitted with the function *fitQTL* where both trait QTL identified in *scanone* were added in the model. The function *refineQTL* was applied and adjusted if the new positions in the ANOVA the model had a greater fit (LOD and *p-values*). The Bayesian credible interval for 5% error rate was implemented as the confidence QTL interval.

### Genome-wide association analysis

The whole population was genotyped using the Axiom *J. regia* 700K SNP array as described in (Marrano *et al.* 2018). Association mapping was performed using the high-quality Poly High Resolution SNPs (335K; for details see (Marrano *et al.* 2018). Quality filtering was performed in PLINK 1.9 (Purcell *et al.* 2007); individuals with less than 90% genotypic data were filtered, along with SNPs having a minor allele frequency less than 5%, resulting in a total of 217K SNPs to apply for analysis. The maximum genotypic call error rate was set at 5%, and SNPs were retained if their genotype frequencies corresponded with Hardy Weinberg expectations (p-value <0.001).

Principal components of SNP markers were obtained by running the mixed linear model (MLM) algorithm in GAPIT. The first component explained 9.39% variation, the second PC explained 6.97% variation, the third PC explained 5.31%, the fourth PC explained 3.41%, and the fifth PC explained 2.63%. The number of principal components (PCs) to include as covariates in the models was defined based on the screeplot (Figure S2**)**.

Genome-wide associations were conducted using the Fixed and Random Model Circulating Probability Unification (FarmCPU) algorithm (Liu *et al.* 2016) and the multi-locus mixed linear model (MLMM) algorithm (Segura *et al.* 2012) in the R package genomic association and prediction integrated tool (GAPIT v3) (Wang and Zhang 2018). The MLMM is a multi-locus mixed linear model (MLM) (Yu *et al.* 2006) where both Q (population structure) + K (kinship matrix) are fitted to the model as random effects, so that type 1 errors are reduced from spurious associations due to relatedness and population structure. A VanRaden kinship matrix (VanRaden 2008), and PC‘s were calculated in GAPIT and added as covariates in the model. FarmCPU is also a multi-locus model developed to control false positives by utilizing markers as covariates in a stepwise MLM to remove markers that are confounding with kinship (Lui *et al.* 2016). This model was set to perform 10 iterations, with four to six PC’s added as covariates, depending upon the goodness of fit in the QQ-plots for each color phenotype, a minor allele frequency threshold of 5%, and the default parameters for bin size. A 5% Bonferroni threshold (2.29 × 10^−7^) was used to assess significance, and Q-Q plots and Manhattan plots were inspected for evidence of inflation. A multiple corrections test (Gao *et al.* 2008) was applied in order to assess SNPs with a less stringent threshold (4.70 × 10^−6^).

### Mapping of candidate genes

Since large LD blocks were observed in the GWAS population, due to high relatedness of individuals, a 100 kb window around significant loci were scanned to identify putative candidate genes. This was determined by examining LD decay plots and there was a large drop in R^2^ between 100-120 kb. Within these genic regions the predicted mapped genes and functional annotation as described in (Marrano *et al.* 2020) were utilized from the new chromosome-level assembly of the ‘Chandler’ reference genome v2.0, found at https://www.ncbi.nlm.nih.gov/assembly/GCA_001411555.2 and annotations found at https://hardwoodgenomics.org/Genome-assembly/2539069?tripal_pane=group_downloads.

### Data availability

All data required to replicate the analyses are available as supplements cited in-text or in Supplemental data files 1-7. Computer vision system methods and macros are available in supplemental (File S1, File S2, File S3) respectively. Output tables from ANOVA in the GWAS population provided in File S4. ‘Chandler’ and ‘Idaho’ genetic maps with phenotypic data are available in supplemental (File S5). Estimated breeding value phenotypes and principal components used in GWAS analysis are available in supplemental (File S6, File S7). Supplemental data file S8 contains raw color score data for both the GWAS population and ‘Chandler’ x ‘Idaho’ population. Supplemental material available at figshare: https://doi.org/10.25387/g3.12276539.

## Results

### Phenotypic variation in pellicle color

In general, ‘Chandler’ produces kernels with an extra-light pellicle color, while Idaho’s kernels are light amber (Figure S1); these differences are consistently observed in both the DFA and CVS analyses. For the ANOVA linear models significant (*P* < 0.0) factors were family, year of harvest, family × year interaction, individual, and age; however age was not a significant factor in the linear models for DFA and L* phenotypes (File S8). For the calculation of adjusted means with ‘Chandler’ × ‘Idaho’ dataset, all terms and their interactions were significant (*P* < 0.0001) (File S4).

**Figure 3:**
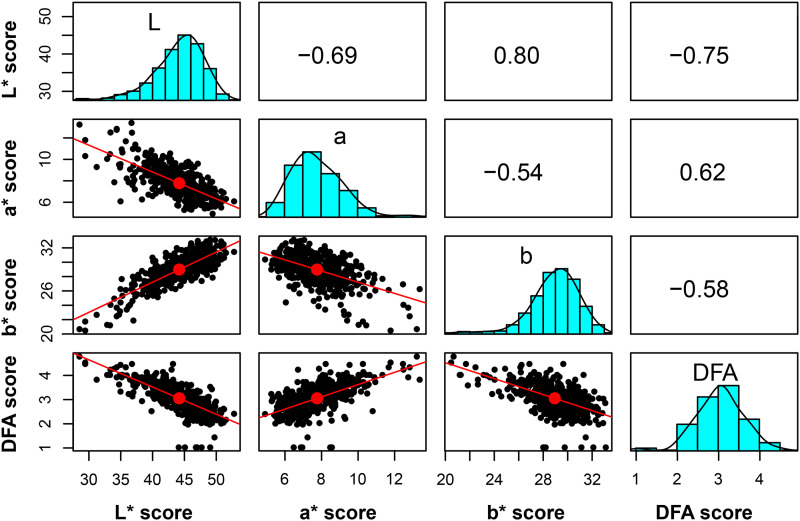
Correlation of traits for walnut pellicle color. Correlation of phenotypic scores (L* a* b* and DFA) based on adjusted means for harvest 2015, 2016 for 528 individual trees. The scale ranging from -1 – +1 indicates level of correlation.

The phenotypic distribution of color scores in this study revealed that the highest frequency of DFA color score is found in the category ‘2’, while the CVS color scores are on a continuous scale (Figure 3). Phenotypic values for L* and b* are highly correlated, 0.80, while a* and DFA* score show a moderate correlation of 0.62 (Figure 3).

### Heritability and breeding value estimates

Pellicle color measurements acquired using the new image-based phenotyping method yielded values of the narrow-sense heritability similar to the DFA scoring system (*h^2^*_DFA_ = 0.56 ± 0.05, *h^2^*_L*_ = 0.58 ± 0.15, *h^2^*_a*_ = 0.60 ± 0.04, *h^2^*_b*_ = 0.53 ± 0.07). ‘Scharsch Franquette’, a selection made in California which is genetically identical to ‘Franquette’, a French variety, is a founder of the UC Davis Walnut Improvement Program, displayed the highest breeding value (47.35) for the lightness L* score. Families that possessed multiple individuals with top L* score breeding values included 10-024 (03-001-977 × ‘Ivanhoe’), 11-030 (04-003-107 × ‘Ivanhoe’) and 09-25 (95-026-16 × 94-019-85) along with founder ‘Waterloo’, and ‘Chandler’ (Table S1).

### QTL mapping

**Figure 4:**
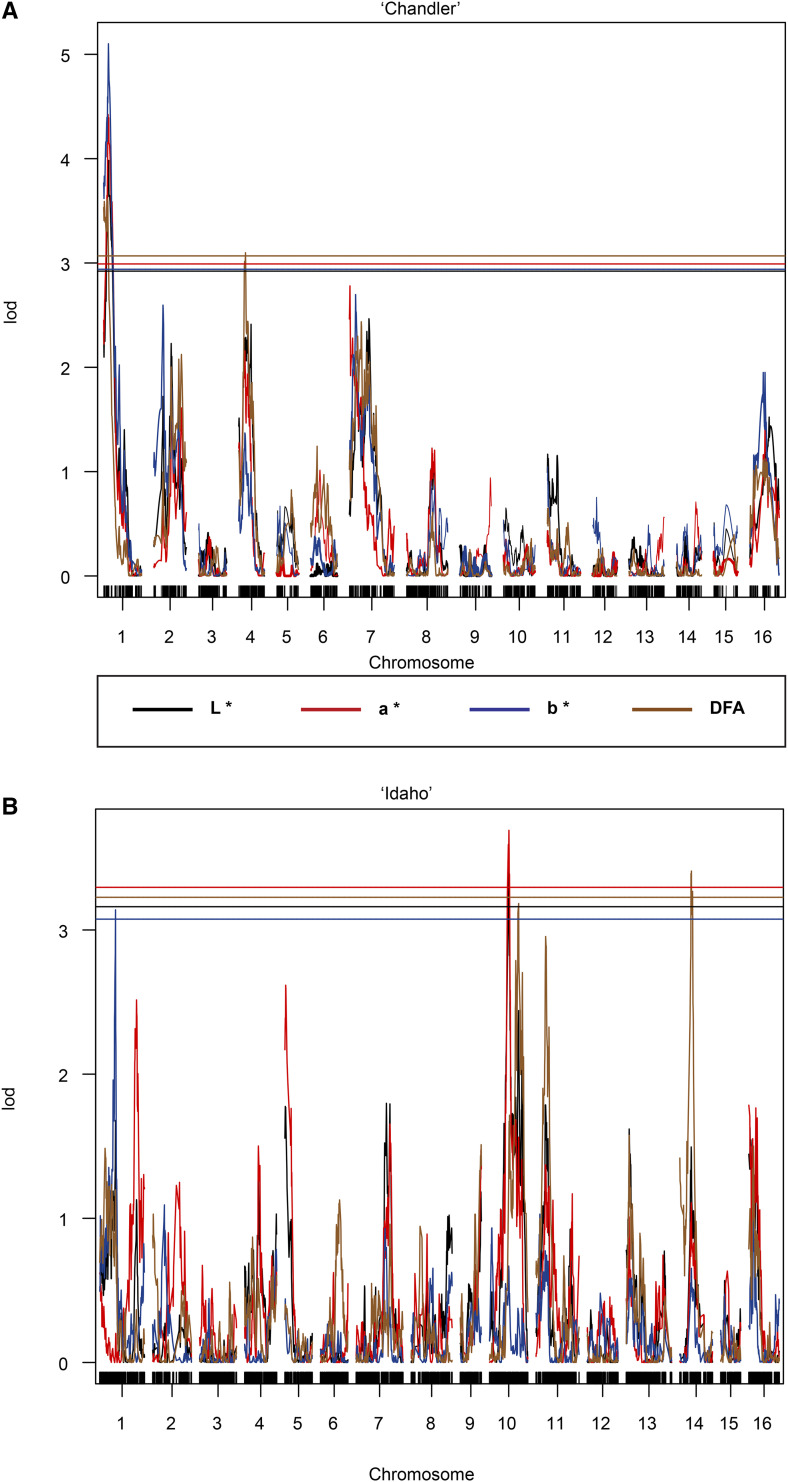
Manhattan plots from QTL mapping of ‘Chandler’ × ‘Idaho’ population. A. SNPs segregating in the ‘Chandler’ population. QTL identified on Chr01 for L*, a*, DFA, scores detected two-year dataset, while b* only detected in 2017, and on Chr04 for the DFA score in both years. B. ‘Idaho’dataset contains QTL identified on Chr10 for L*, a*, and DFA averaged scores over 2016 and 2017, and QTL on Chr14 for the DFA score in 2017.

Color scores often had overlapping regions of significant marker trait associations (Table 1, Figure 4). For the ‘Chandler’ map, QTL explaining L* and a* score phenotypes were identified on Chr01 and Chr16, where only on Chr01 was a stable QTL for both 2016 and 2017 thereby explaining 11.62% of variance for L* and 10.53% for a* (Figure 4, Figure S3). Additional QTL’s for a* score phenotype in the ‘Chandler’ map were detected on Chr07 in the year 2016 only (Figure S4). For the DFA score in the ‘Chandler’ map, QTL were located on Chr01, in an overlapping interval with L* and a* (Figure 4), and Chr04 (Figure S5). In ‘Idaho’ map, a QTL for both L* and a* score phenotypes was detected on Chr10 in both 2016 and 2017 explaining 9% of the variance for those traits. A second QTL on Chr10 was identified for the DFA score (Figure 4). In addition, a QTL for DFA scores was detected on Chr14 in correspondence with the QTL identified for the L* and the a* phenotypes (Figure S6). For the b* score, two QTL were identified on Chr01 in both populations ‘Chandler’ and ‘Idaho’, and they overlapped for some of their length (‘Chandler’ QTL interval 2,966,643 – 9,763,773 bp; ‘Idaho’ QTL interval 8,826,101 – 14,691,279 bp) (Figure 4, Figure S7).

**Table 1:** Results from QTL mapping for 168 individuals in the ‘Chandler’ × ‘Idaho’ mapping population for different color phenotypes. *Interval* was determined by Bayes 95% Credible Interval, *Pvalues* converted from LOD score cutoff determined from 1000 permutations of phenotypic data, *R^2^* is the variance explained for QTL; if two years of data then R2 taken on multi-year data. *Year of detection* includes a single year of detection, or in both years 2016/2017

Trait	Population	Chr	Pos (cM)	Interval	Physical Position (bp)	Pvalue	R^2^	Year of detection
L*	Chandler	1	9.58	5.00 - 18.00	4620011	4.87^−6^	0.107	17
L*	Chandler	1	10.18	2.98 - 22.59	4756788	9.07^−6^	0.116	16, 17
L*	Chandler	16	27.27	16.24 - 36.89	20234419	2.58^−5^	0.090	17
L*	Idaho	10	59.11	41.25 - 65.66	14732679	3.53^−5^	0.090	16, 17
L*	Idaho	14	34.02	27.00 - 42.00	6515088	1.11^−4^	0.076	17
a*	Chandler	1	9.60	5.39 - 18.00	4532719	3.63^−6^	0.103	16, 17
a*	Chandler	7	1.19	0 - 70.00	993462	9.29^−6^	0.105	16
a*	Chandler	16	27.27	16.24 - 36.89	20234419	1.16^−4^	0.066	17
a*	Idaho	10	59.11	41.25 - 65.66	14732679	9.14^−5^	0.090	17
a*	Idaho	14	35.21	27.00 - 42.00	6764116	4.29^−5^	0.087	16, 17
b*	Chandler	1	9.57	2.98 - 22.59	4620011	6.29^−7^	0.130	17
b*	Idaho	1	48.12	35.10 – 53.00	13073793	7.16^−5^	0.082	16, 17
DFA	Chandler	1	9.96	0 - 10.00	4223640	1.88^−5^	0.096	16, 17
DFA	Chandler	4	13.69	8.93 - 25.00	17907379	8.09^−5^	0.087	16, 17
DFA	Idaho	10	88.89	73.00 - 104.00	31577799	5.33^−5^	0.086	17
DFA	Idaho	14	38.00	28.00 - 41.76	6764116	3.63^−5^	0.092	17

### Genome-wide association mapping

**Figure 5:**
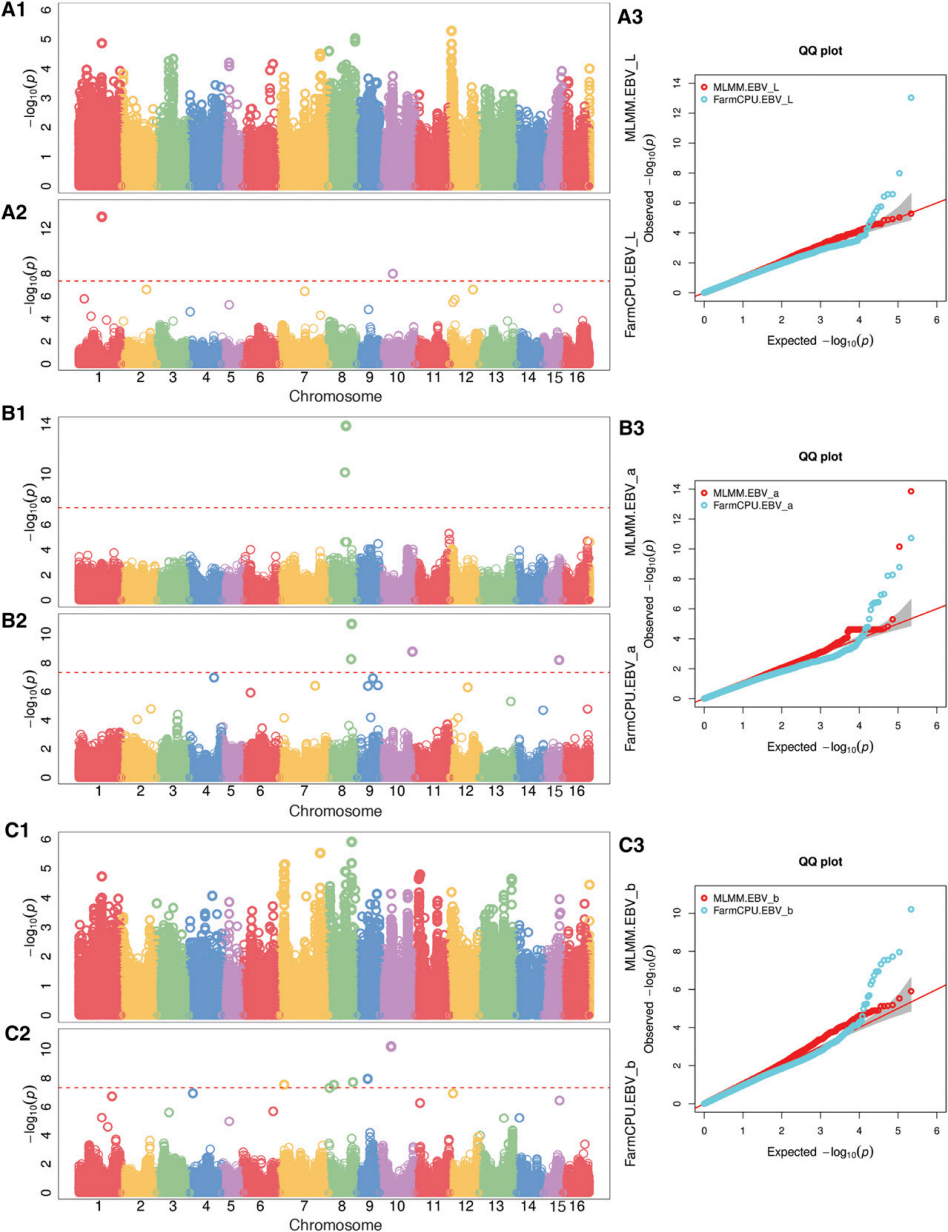
Manhattan plots from genome-wide association analysis with CVS phenotypes. A1. L* results with MLMM model A2. L* results with FarmCPU model. A3.L* QQ-plots for both models. B1. a* results with MLMM model B2. L* results with FarmCPU model. B3. a* QQ-plots for both models. C1.b* results with MLMM model B2. b* results with FarmCPU model. B3.b* QQ-plots for both models. SNPs that did not anchor to any chromosome during the assembly were left as undefined in the orange after Chr16.

Principal component analysis revealed four to six principal components which explained SNP variation for the 31 full-sib families in the Walnut Improvement Program, excluding ‘Chandler’ × ‘Idaho’ (Figure S2). See Figure 5 for an overview of Manhattan plots for CVS phenotypes. The most significant marker trait association for the L* score was on Chr01 with a MAF of 0.20 (Table 2). There were significant SNPs detected for both L* and b* scores that were identical (Chr01 and Chr12). For the a* score, the most significant marker trait association was found on Chr08, while significant trait-loci correlation were also identified on Chr10, Chr11, Chr14, Chr15, Chr16 with different models. For the b* score, both the MLMM and FarmCPU models detected significant associations on Chr07 and Chr08. Additional associations for the b* score were found on Chr09 and Chr10, with many other small QTL just below threshold (Chr01, Ch04, Chr11, Chr12). For the DFA scores, a significant marker trait association on Chr08 was detected with both MLMM and FCPU models (Figure S8). Significant associations were identified in similar chromosomal regions for both the CVS and DFA scores, even though many of them did not reach the significance level of 2.29 × 10^−7^ (*i.e.*, Chr 01, Chr09 and Chr13, Chr16) (Figure S8).

**Table 2:** Most significant marker-trait associations from genome-wide association scan of (n = 528)

Trait	Chr	Position (bp)	Pvalue	*^a^*MAF	*^b^*R^2^	Effect (est)
L*	1	**24588560**	2.05^−15^	0.20	0.05	−1.02
L*	4	3580183	5.48^−8^	0.16	0.02	0.73
L*	5	7700218	2.73^−11^	0.10	0.07	−1.08
L*	8	28491012	2.49^−11^	0.07	0.04	−1.28
L*	9	14110666	8.11^−8^	0.47	0.07	0.63
L*	12	1886325	5.18^−6^	0.14	0.05	0.66
L*	12	3406040	2.66^−6^	0.47	0.06	0.88
L*	15	15274698	9.08^−10^	0.09	0.02	1.05
a*	1	37670750	7.41^−7^	0.11	0.05	−0.30
a*	8	24731099	5.22^−8^	0.09	0.07	−0.81
a*	8	18643646	1.64^−14^	0.10	0.04	−0.28
a*	10	34936156	7.66^−9^	0.45	0.04	−0.25
a*	11	36386668	5.44^−6^	0.05	0.03	0.23
a*	14	3094777	5.70^−7^	0.28	0.04	−0.26
a*	15	16729948	3.28^−9^	0.07	0.01	0.45
a*	16	26668826	2.39^−5^	0.09	0.04	0.26
b*	1	**24588560**	1.15^−12^	0.20	0.05	−1.02
b*	1	35482903	1.83^−7^	0.41	0.04	0.46
b*	7	5027345	7.56^−6^	0.06	0.05	−0.24
b*	7	43498845	2.61^−6^	0.09	0.06	0.44
b*	8	24680376	5.88^−6^	0.30	0.06	0.31
b*	9	11456318	7.44^−7^	0.24	0.06	−0.29
b*	10	12202442	1.03^−8^	0.43	0.04	0.38
b*	11	5382614	1.62^−5^	0.16	0.02	−0.35
b*	12	3406040	6.27^−8^	0.47	0.06	0.88
b*	13	26311574	6.23^−6^	0.09	0.01	−0.24
b*	13	34566210	2.73^−7^	0.06	0.05	0.63
b*	14	3126302	6.48^−9^	0.29	0.04	0.53
DFA	6	13137859	8.99^−9^	0.36	0.006	0.26
DFA	7	51065074	4.70^−11^	0.14	0.08	−0.13
DFA	8	28588006	4.25^−6^	0.07	0.02	0.17
DFA	9	14771377	1.62^−6^	0.43	0.03	0.05
DFA	12	25920027	4.26^−7^	0.49	0.05	−0.07
DFA	13	38230982	1.11^−9^	0.49	0.01	0.07
DFA	16	26455353	3.47^−7^	0.14	0.04	0.09

a MAF minor allele frequency.

b R^2^ variance explained by significant SNP, P.value significance from Bonferroni taken from L* = model with 4 PCs, a* = model with 4 PCs, b* = model with 6 PCs, DFA = model from 5 PCs.

**Figure 6:**
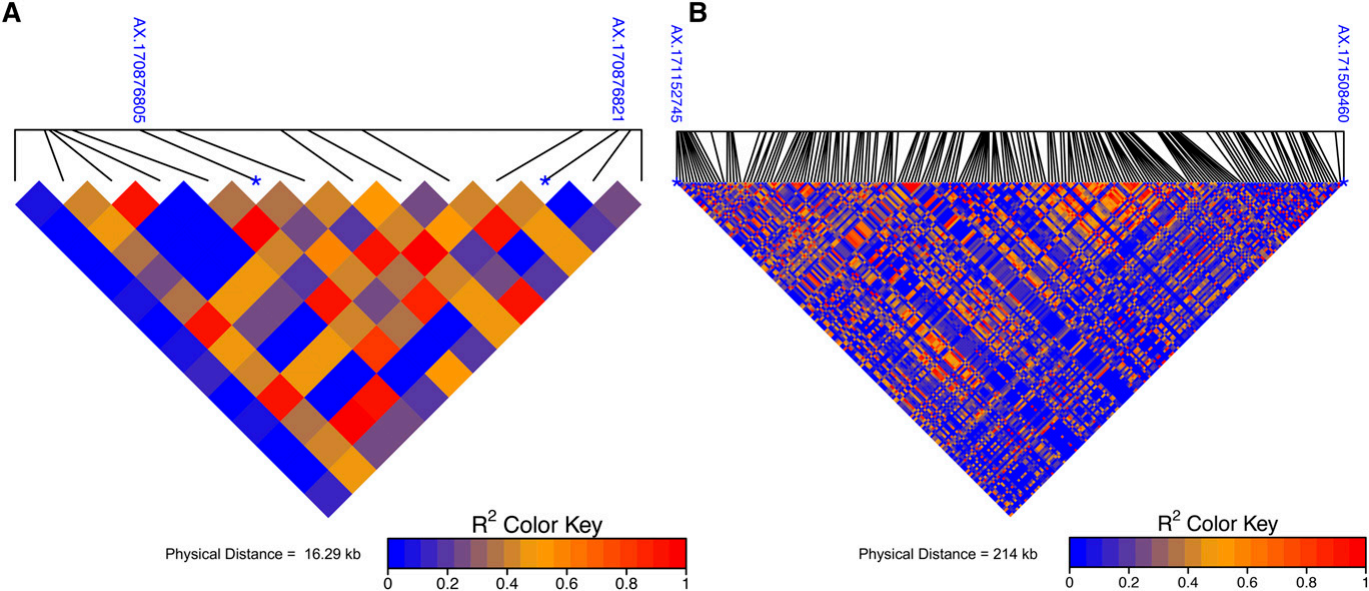
Pairwise LD between associated SNPs for DFA and CVS color phenotypes. A. Chr01 SNP AX.17087682 associated with DFA phenotype, and the SNP AX.170876805 associated with L* phenotype have a physical distance of 16.29 kb and LD measure of r^2^ = 0.36. B. Chr16 SNP AX.171152745 associated with DFA phenotype and SNP a* AX.171508460 have a physical distance of 214 kb and LD measures r^2^ = 0.62.

The SNP associated with the DFA scores were in moderate LD with SNP associated with the L* scores on Chr01 (r^2^ = 0.37) and with SNP associated with the a* score on Chr16 (r^2^= 0.63). Pairwise LD measures between these SNPs associated with DFA and L* scores and SNPs associated with DFA and a* scores are visualized to show close proximity to each other 16.29 kb and 213 kb respectively (Figure 6).

### Co-localization of marker trait associations in QTL mapping and GWAS

**Figure 7:**
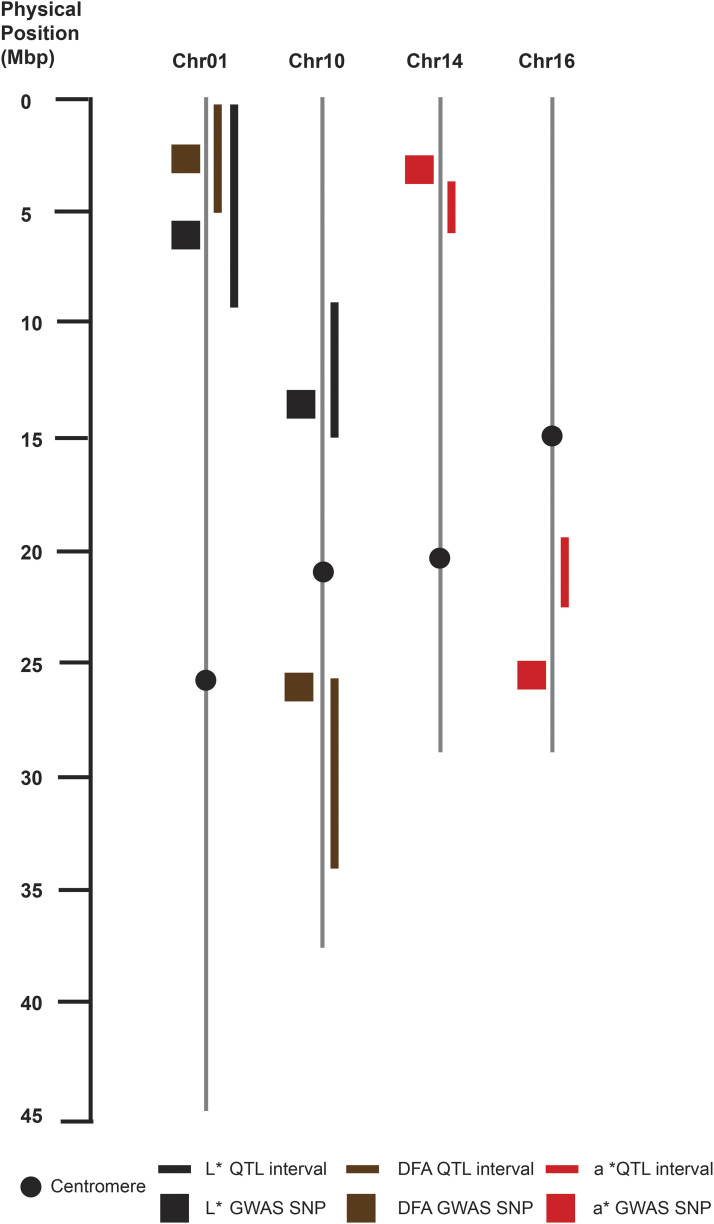
Co-localization of QTL mapping and genome-wide association results. Associations for the L*, DFA and a* phenotypes on Chr01, 10, 14, 16 displayed with corresponding colored boxes or lines.

In many cases there were overlapping regions between marker trait associations detected in both QTL mapping and GWAS (Figure 7). For Chr01, both QTL intervals in association with L* score and DFA score detected in ‘Chandler’ map (220,484 - 9,763,773 bp) and (1,495,995 – 4,819,547 bp) respectively, and SNPs identified from GWAS within those intervals (5,824,300 bp) and (2,674,553 bp) respectively overlapped. Chromosome 10 displayed two co-localized regions for both associations found for L* and DFA score in the ‘Idaho’ map and the GWAS; L* score phenotype associated with SNP at (14,011,277 bp) fell within (9,513,973 – 15,033,186 bp) QTL region, and DFA score associated with SNP at (26,815,371 bp) lied within the (26,206,323 – 35,601,472 bp) interval. Due to a 10cM marker gap on Chr14 in ‘Idaho’map, the nearest SNP to the a* QTL interval began at (4,615,740 bp), which was 1.5 Mbp away from the significant SNP identified with the GWAS at (3,094,777 bp). Lastly, on Chr16 the SNP (26,668,826 bp) associated with the a* score lies 4.47 Mbp (22,190,531 bp) away from the end of the QTL interval identified in ‘Chandler’ map.

Similarly, (Marrano *et al.* 2019)) performed a GWAS analysis on 584 walnut trees of the UC Davis Walnut Improvement Program utilizing 30 years of historical data based upon the DFA scoring method. There were two marker trait associations; Chr07 and Chr09 which co-located with three marker trait associations in this study for DFA and L* scores, and were in moderate LD (r^2^ = 0.54, 0.64, 0.50) respectively (**Table S2**).

### Complete L*, a*, b* information for putative gene functions

**Figure 8:**
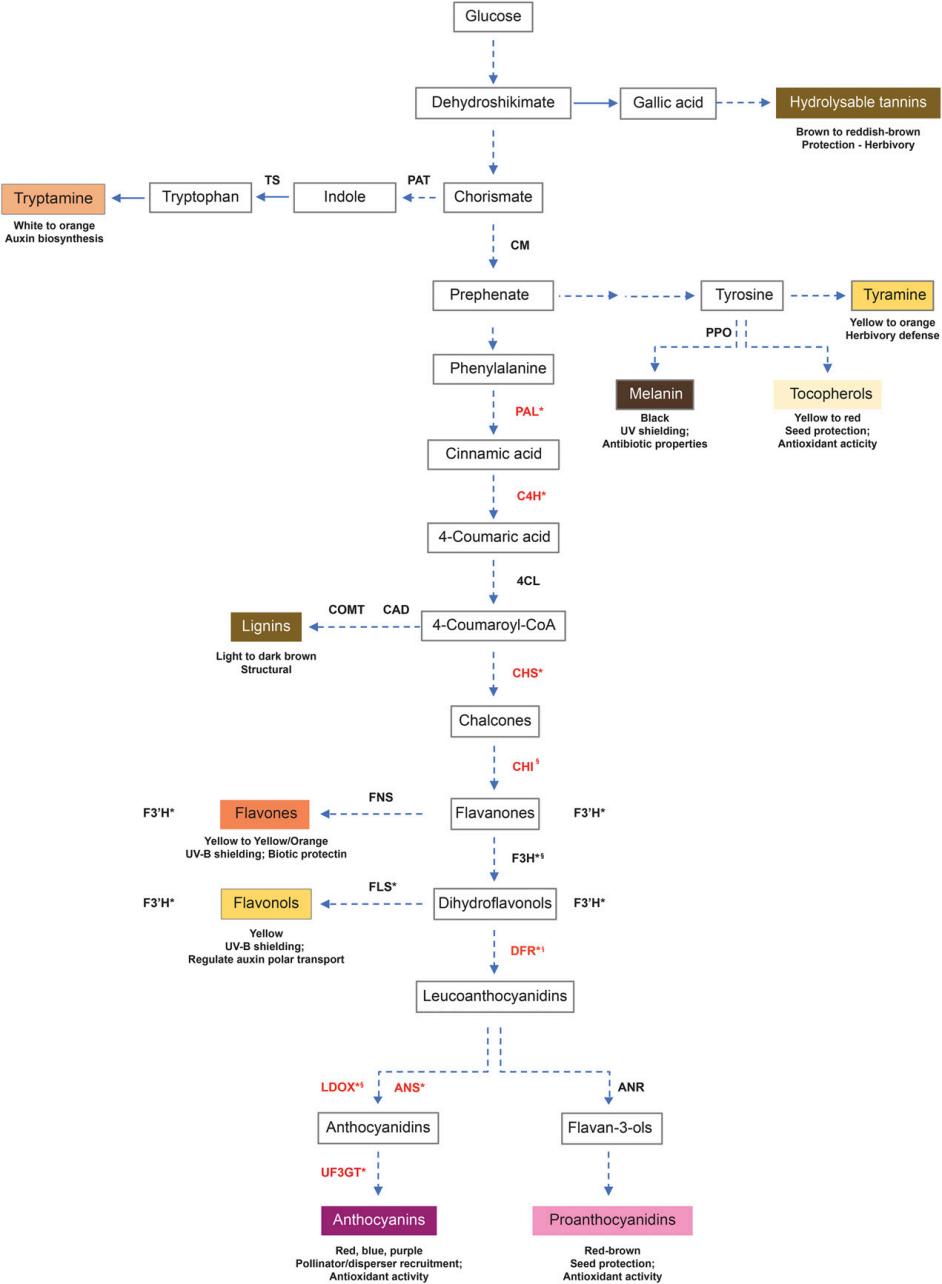
Flavonoid biosynthesis overview. Shown is a summary of flavonoid biosynthesis, including the shikimate, aromatic amino acid, phenylpropanoid and flavonoid biosynthesis pathways. Gene abbreviations in black were found to be associated with SNPs in this study. The expression of genes in red are known to be regulated by MYB1 and/or MYB113 transcription factors, but not associated with the SNPs reported in this study.

Six hundred, twenty-nine (629) putative candidate genes were identified within 100 kb windows surrounding the significant SNPs (Table S3). These putative candidate genes are predicted to have functions related to abiotic stress, pathogen resistance, hormone signaling and the biosynthesis of pigmented metabolites (Table S3, Table 3, Figure 8). Three putative gene functions were identified in association with SNPs on Chr01. The first of these gene functions is the transcription factor MYB113 which is associated with the L* score. The second gene function appears to encode cinnamoyl alcohol dehydrogenase (*CAD*), which is associated with the b* score. Lastly, the third gene function which lies on Chr01 encodes tryptophan synthase (TRP), which is also associated with the b* score. One gene function (transcription factor MYB1) associated with the DFA score was identified on Chr07. For the a* score, on Chr10 flavonol synthase/flavanone 3-hydroxylase (*FLS*) was identified to be associated with a SNP, as well as on Chr11 chorismate mutase (*CM*). Two putative gene functions were identified on Chr12; the first of these genes was associated with both an L* and b* score, and encoded 4-coumarate—CoA ligase while the second gene encoded flavonoid 3′-monoxygenase, was associated with a DNA score. Four gene functions were identified to be associated with SNPs on Chr13; the first gene function is anthranilate phosphoribosyltransferase (PAT) which was associated with a b* score, the second gene function was also associated with a b* score and encoded caffeic acid 3-O-methyltransferase (*COMT*), and the final two gene functions on Chr13 both encoded *FLS* genes, were both associated with L* scores (Table 3).

**Table 3:** Gene products for putative candidate genes contributing to pellicle color

Chr	Position	Gene start	Gene stop	Gene ID	Transcript ID	Putative gene name	Trait
Chr01	5824300	5746605	5753513	Jr01_08300	XP_018816729.1	Transcription factor MYB113	L*
Chr01	13073793	13032403	13039428	Jr01_15230	XP_018824948.1	Cinnamoyl alcohol dehydrogenase (CAD)	b*
Chr01	42151069	42158912	42166648	Jr01_31290	XP_018827345.1	Tryptophan synthase (TS)	b*
Chr07	51065074	50989265	50992246	Jr07_37390	XP_018821900.1	Transcription factor MYB1	DFA
Chr10	34936156	34824830	34829124	Jr10_23440	XP_018808783.1	Flavonol synthase/flavanone 3-hydroxylase (FLS/F3′H)	a*
Chr11	36386668	36459446	36461883	Jr11_30250	XP_018833669.1	Chorismate mutase 2 (CM)	a*
Chr12	3406040	3421907	3426094	Jr12_02860	XP_018830336.1	4-coumarate–CoA ligase 1 (4CL)	L*, b*
Chr12	25747726	25971475	25974144	Jr12_17130	XP_018860732.1	Flavonoid 3′-monooxygenase (F3H)	DFA
Chr13	26311574	26345153	26350078	Jr13_21620	XP_018819013.1	Anthranilate phosphoribosyltransferase (PAT)	b*
Chr13	34566210	34564434	34567086	Jr13_26200	XP_018817142.1	Caffeic acid 3-O-methyltransferase (COMT)	b*
Chr13	35292203	35225821	35228439	Jr13_26690	XP_018817156.1	Flavonol synthase/flavanone 3-hydroxylase (FLS/F3′H)	L*
Chr13	35292203	35246890	35252142	Jr13_26700	XP_018820940.1	Flavonol synthase/flavanone 3-hydroxylase (FLS/F3′H)	L*

## Discussion

The use of semi-automated and high throughput phenotyping of walnut pellicles uncovered novel SNPs associated with color in walnut pellicles. The CVS was able to deconstruct the three main color components contributing to trait variation which can be used as a fast and reliable method for data collection and processing walnut pellicles.

By combining two complementary approaches, QTL mapping and genome-wide association, we performed genetic analysis for pigmentation in walnut pellicles clearly indicating a quantitative nature of the trait by observing a few large effect loci, and many small effects loci contributing to trait variation. In our QTL mapping study with a population size of about 200, only Chr01 and Chr07 harbored QTL explaining greater than or equal to 10% total variance, while most QTL found explained between 6–9% of the variance, and the average genetic variance explained by SNP was 5%. In our GWAS, with a population size of about 530, there were several small and one or two large effect QTL for each color phenotype.

For the QTL mapping analysis, the ‘Idaho’ genetic map which was constructed in (Sideli *et al.* 2020) was found to have nearly a twofold increase range in map size of ‘Chandler’ genetic map. Shorter genetic maps of female parent in walnut and other species has also been observed (Luo *et al.* 2015; Kefayati *et al.* 2019; Marrano *et al.* 2019) thereby suggesting a lower recombination rate in the female parent of walnut. ‘Chandler’ was also found to have many homozygous regions which further supports lower recombination (Marrano *et al.* 2020). On Chr01 of ‘Chandler’ genetic map, multiple QTL were identified in the same genomic region for all color phenotypes. This can suggest that the color determination is detecting the same differences contributing to pigmentation due the correlation between color scores; L* (lightness) was found to be in high association with the b* (amount of yellow), and a* (amount of red) in moderate association with DFA. The DFA scoring phenotype has low resolution power resulting in a QTL curve that is less significant than the other three color phenotypes. Consequently, it is less sensitive because it lacks the ability to discriminate specific color components L*, a*, b* simply because it encompasses all three measurements. The fact that the L* and a* were found to have overlapping regions of association is due to the fact that in the biosynthetic pathway, flavonol synthase/ (FLS/F3H) is bifunctional to perform different reactions forming flavonols (FLS) or proanthocyanins/anthocyanins (F3H) (Lukačin *et al.* 2003), all which are pigmented in a different way suggesting a complex regulation for production of flavonols. Further exploration is needed to rule out possible pleiotropy.

For the GWAS analysis we tested two models, FarmCPU and MLMM, in order to account for population structure and/or kinship in our population as to reduce any false positive or false negative associations. As applied in the FarmCPU model, PCs will help account for population structure, while for MLMM model, both PCs and a kinship matrix is applied to account for levels of relatedness among individuals (Yu *et al.* 2006). The FarmCPU method has a higher detection for marker trait associations and the MLMM is known to miss associations if they are related with population structure (Cui *et al.* 2018). In our study we detected QTL that overlapped with methods (QTL mapping and GWAS) and models, and each method was able to detect additional QTL with large effects. Here, FarmCPU method was more sensitive in that it detected more marker-trait associations with greater effects than the MLMM method. However, a plausible explanation is that some of these associations can be related to population relatedness, since they were not detected by the MLMM, which explicitly accounts for levels of relatedness using the Q and K matricies as covariates.

Several candidate genes acting in pathways linked with the production of pigmented metabolites in the flavonoid biosynthetic pathway were identified by association with SNPs generated by the GWAS and QTL analysis. Two putative genes in early flavonoid biosynthesis, *CM* and *FLS/F3H* were associated with the a* score (9.5, 9.4 respectively), indicating that these genes may contribute to a slight reddish/brown pigmentation. CM is involved in the biosynthesis of phenylalanine and tyrosine as well as their derivatives, the phenylpropanoids, lignins, flavonoids and anthocyanins, most of which are pigmented (Cotton and Gibson 1965; Cotton and Gibson 1968). *FLS/F3H* is a bifunctional enzyme (Lukačin *et al.* 2003); F3H generates the dihydroflavonols which are precursors to anthocyanins and red-brown proanthocyanidins (Britsch *et al.* 1981; Holton *et al.* 1993). Similarly, the putative genes *PAT*, *TS*, *4CL*, *CAD* and *COMT* were associated with the b* score (26.5, 27.1, 27.6, 27.5, 27.4, respectively), suggesting that these genes promote yellow color. *PAT* and *TS* both act in the biosynthesis of tryptophan, a light yellow AAA and its yellow/orange derivative tryptamine (Lovenberg *et al.* 1962; Baxter and Slaytor 1972; Gibson *et al.* 1972). *4CL* yields a precursor for brown pigmented lignins and the rest of the flavonoid biosynthesis pathway (Lindl *et al.* 1973; Gross and Zenk 1974). *COMT* and *CAD* both act in modifying lignin precursors (Poulton *et al.* 1976; Wyrambik and Grisebach 1979; Sarni *et al.* 1984; Gowri *et al.* 1991). As previously described *4CL* and *FLS/F3H*, also correspond to association with the L* score (41.5, 42.0 respectively), suggesting that these genes are associated with a pigmentation of the pellicle; bifunctional *FLS/F3H*- *FLS* desaturates dihydroflavonols to yield yellow/brown flavonols (Forkmann *et al.* 1980; Charrier *et al.* 1995). Finally, the putative gene *F3′H* was associated with the DFA score (2.2), indicating a possible diversity of colored pigments. *F3′H* hydroxylates flavanones, dihydroflavonols, flavones and flavonols to increase the diversity of each of the aforementioned categories of yellow flavonoids (Forkmann *et al.* 1980; Brugliera *et al.* 1999).

The results from this study will enable molecular breeders to develop kompetitive allele specific pcr (KASP) markers from the most significant SNPs, assisting in efficient selection at the seedling stage rather than the selection process over a 5-10 years period requiring trees to reach sexual maturity. Early selection of progeny will dramatically increase the efficiency in developing new cultivars. Due to the fact that walnut growers are compensated based on human grader’s perception of color, an improvement to the CVS algorithm would include human input factors such as discrimination for veining, speckling or size can be considered.

For further investigation, the creation and evaluation of genomic prediction models will enable the integration of all the small QTL effects to predict progeny with lighter pellicles, as the industry demands. To solidify results pertaining to the putative candidate genes for flavonoid production, an experiment setup to clone each gene and express them in bacteria would verify gene function and identify the origin of the chemical reactions occurring in response to developmental stages, environmental stress or autophagy.
